# Root-derived allelochemicals from *Moringa oleifera* regulate germination and early seedling growth in New Zealand pasture, native, and weed species

**DOI:** 10.1080/15592324.2026.2644120

**Published:** 2026-03-17

**Authors:** Blair Moses Kamanga, Paul Barrett, Donita L. Cartmill, Craig McGill, Andrea Clavijo McCormick

**Affiliations:** a School of Agriculture and Environment, Massey University, Palmerston North, New Zealand

**Keywords:** Allelochemicals, target species, inhibition, metabolomics, species interaction, stimulatory effects, provenances

## Abstract

*Moringa oleifera* L. (moringa) is not currently commercially cultivated in New Zealand, but there is growing interest in its integration into farming systems. However, there are questions regarding its ecological impacts. One area of interest is its allelopathic traits, which may affect native and other introduced plant species. Hence, this study evauated the effects of root-derived moringa allelochemicals on the germination and seedling growth of pasture, weed an native species and employed untargeted metabolomics to identify potential bioactive compounds responsible for the observed effects, and their biosynthetic pathways. . Root extracts from two moringa provenances were assessed across concentration gradients (1–100%) in laboratory bioassays, while potted plant experiments were conducted under semi-controlled conditions to evaluate **allelochemical-mediated plant–plant interactions**.. Metabolomics analysis revealed several bioactive compounds with potential allelopathic activities dominated by phenylpropanoids and benzenoids, with relatively small contributions from organoheterocyclics, oxygenated compounds, and organic acids. Statistical analyses demonstrated a dose-dependent and species-specific response to moringa root allelochemicals, with white clover, a key pasture species in New Zealand, being the most adversely affected. Therefore, the utilisation of moringa within white clover pastoral systems may not be advised. Isolating and testing individual compounds will be essential for determining how moringa may influence plant growth and population dynamics in New Zealand pastoral and native ecosystems.

## 1. Introduction

Plant roots constitute a significant portion of plant biomass and perform essential functions, such as anchorage, and the acquisition, uptake and storage of soil-based resources (mainly water and ions). However, roots are also involved in the production, storage and release of primary and secondary metabolites.^1-3^ Primary metabolites are essential for plant growth and reproduction and have regulatory properties, whereas secondary metabolites play vital roles in plant‒plant interactions and ecosystem dynamics.^4^^,^^5^ They are released into the soil through root exudation, leaching, volatilisation, and decomposition of root tissues, which impacts nearby plants and soil biota.^6^ The chemical compounds that elicit a response in another plant or organism are collectively known as “allelochemicals” and may have positive or negative impacts on the growth, reproduction or behaviour of the receiver organisms.^7-9^

Root allelochemicals represent a diverse group of bioactive compounds with the potential to improve crop growth through their positive effects on physiological activities, nutrient uptake, and plant stress tolerance mechanisms.^10-12^ Conversely, certain root allelochemicals have inhibitory effects on the germination and growth of neighbouring plants.^13^^,^^14^ While the inhibitory traits of certain root compounds could be beneficial for weed suppression in an agricultural system, they could also pose serious challenges in managed and natural ecosystems by inhibiting other agricultural and important native species and further disrupting the ecological balance.^13^^,^^15^ This complex nature of allelochemicals emphasises the need for a nuanced understanding and application of these traits in agricultural and ecological contexts. Adding to this complexity, receiving plants are known to react differently to allelochemicals released by species they coevolved with versus not, and their concentration determines the overall effect of a given chemical compound or blend.^16^

In response to climate change and resource availability pressures, producers are increasingly exploring the establishment of resilient, multi-purpose perennial species such as *Moringa oleifera* L. (moringa) within cropping and pasture systems. This approach aims to diversify production (e.g., animal feed, human consumption, biofuel), while enhancing structural complexity and biodiversity.^17-20^ Moringa is native to the Indian subcontinent; however, it has been cultivated worldwide across a range of climates, predominantly in tropical and subtropical regions.^21^ While moringa is neither native to nor commercially cultivated in New Zealand, there is growing interest in its integration into local farming systems.^22^ However, there are questions regarding its potential ecological impact, especially any allelopathic traits that could impact established native and introduced pastoral species.^23-25^

New Zealand pastoral systems are dominated by high value forage species such as perennial ryegrass (*Lolium perenne* L.) and white clover (*Trifolium repens* L.), which support pasture productivity, persistence, and biological nitrogen fixation.^26^^,^^27^ Since these species form the foundation of temperate pasture ecosystems, any allelochemical interference arising from the introduction of other species such as moringa requires careful assessment. Studies have demonstrated that legumes are more sensitive to allelopathic compounds,^28^ whereas ryegrasses provide a robust indicator of early competitive responses within a mixed sward.^27^ Therefore, these commonly sown species were selected for growth assays to evaluate their susceptibility to moringa root-derived allelochemicals and to determine the potential compatibility of moringa within temperate pastoral systems.

Research has shown that different parts of a plant, including flowers, fruits, seeds, and roots, produce unique bioactive compounds with specific compositions and properties.^29^^,^^30^ While moringa leaf extracts (MLEs) have received much attention for their bioactive compounds, including phenolic acids, flavonoids, tannins, and glucosinolates) that are known to suppress seed germination and early seedling growth,^11^^,^^14^ moringa root extracts (MREs) remain insufficiently studied,^11^ despite roots being important sources of secondary metabolites.^31^ Since the concentration of these metabolites varies with genotype and in response to the environment where the plant is grown, two moringa provenances were used to capture potential differences in allelopathic potency. Provenances from hotter or drier regions typically accumulate higher levels of secondary metabolites, whereas those from more moderate climates often contain lower or more balanced concentrations.^32-34^ Comparing these provenances could therefore provide insights on how moringa allelochemicals may vary in response to different environments.

This study was conducted to assess the chemical composition of MREs from moringa plants from two provenances in India and the allelopathic effects of different concentrations of MREs on the germination and seedling growth of New Zealand forages, including perennial ryegrass (*Lolium perenne* L.) and white clover (*Trifolium repens* L.). Weed species assessed, included fathen (*Chenopodium album* L.) and crabgrass (*Digitaria sanguinalis* L. Scop.), and a native species i.e., mānuka (*Leptospermum scoparium* J.R.Forst. et G.Forst.) was also tested. Furthermore, pot-based plant‒plant interference assay (seeds grown together with moringa plants on the same soil) were conducted. We hypothesised that root-derived allelochemicals from moringa modulate germination and growth trajectories of pasture, native, and weed species in species-specific and concentration-dependent ways. By identifying bioactive compounds in moringa roots and evaluating their allelopathic effects, we aim to advance our understanding of the ecological interactions that underpin the sustainable integration of moringa into New Zealand agricultural systems.

## 2. Materials and methods

### 2.1. Experimental site and plant materials

Plants were grown under greenhouse conditions between June and November 2024 at the Plant Growth Unit (PGU), Massey University (−40.37807°S, 175.61362°E). Temperatures were maintained between 24–27°C (±3°C), relative humidity between 65−80%, and plants were exposed to natural light. Seeds of two moringa provenances (PKM-1) were used for the cultivation of donor plants, i.e., provenance_t from Tamil Nadu (South India) and provenance_g from the villages of Deoria District, Uttar Pradesh, (North India). The target species were mānuka seeds collected from the Pasture and Crop Research Unit (PCRU), Massey University (2024), ‘Nui’ perennial ryegrass (ryegrass) and ‘Kotuku’ white clover seeds (2024) were sourced from the AsureQuality Seed Laboratory (New Zealand) and weed species seeds (fathen and crabgrass) were collected in Christchurch, New Zealand, in 2022. Allelopathic effects were assessed using two complementary approaches: root extract assays on steel blue germination paper and pot-based plant‒plant interference assays.

### 2.2. Root sample preparation for metabolomics analysis

Moringa root tissues (100 g) were collected from 120-day-old plants, snap-frozen in liquid nitrogen, freeze-dried, and ground (≈50–150 µm) before metabolite extraction. Metabolomic analysis followed the procedure of Barrett et al.^35^ with minor modification. Briefly, root samples were collected between the months of July and November 2024 from moringa plants raised in a glasshouse at 25±3 °C. Approximately 200 mg of root tissues were collected and folded in aluminium foil, snap-frozen in liquid nitrogen before being transferred to −80 °C for storage. All samples were freeze dried then stored for 2 weeks at −20 °C prior to grinding to approximately 50–150 µm particle size before extraction. At least 50 ± 2.0 mg of ground sample were weighed into 2 mL Eppendorf microcentrifuge tubes, each with a 2.5 mm glass bead and extracted with 800 µL of pre-chilled chloroform CHCl_3_: MeOH (1:1 v/v). Samples were homogenised for 5 mins at 25 Hz sec^−1^ using a Retsch MM400 Mill/Tissue Lyser, then stored for 1 hr at −20 °C.

Then, 400 ml of Ultra High Performance Liquid Chromatography (UHPLC) grade H_2_O was added to each tube, similarly homogenised for 5 mins, and centrifuged (Sigma 1–14K microcentrifuge) at 4 °C and 11000 RPM for 15 mins to create a biphasic layer. For each sample, 250 µl of the upper layer was pipetted into a 2 mL microcentrifuge tube for reverse phase C18 analysis and a second 250 µl aliquot taken for hydrophilic interaction liquid chromatography (HILIC) analysis. A final 250 µl aliquot from each treatment was added to a 150 ml plastic tube, ultimately pooling every sample of each treatment into a homogenous mix, then sub-aliquots (250 µl) of this mix were transferred into microcentrifuge tubes to use as quality controls (QC’s). All tubes were then dried down under a continuous flow of N_2_ (3.5 L min^−1^) and at 40 °C for 55 min using a BT Lab Systems sample concentrator, then immediately stored at −80 °C until reconstitution.

Reconstitution solvents which included an internal standard (MSK-QC-KIT), (Cambridge Isotope Laboratories Inc.) at a concentration of 10 µl ml^−1^ were prepared for C18 analysis in ACN: H_2_O (1:9, v/v) and for HILIC in ACN: H2O (1:1, v/v). Immediately prior to UHPLC-MS (Mass Spectrometry) analysis, all samples plus 7 quality controls (QCs) were reconstituted by adding 250 µl of solvent. Samples were vortexed until dissolved and transferred by pipette into a 250 µl glass insert in a clear autosampler vial, capped, kept dark and chilled until loading. The sequence was five vials of reconstitution solvent only (blanks),1 amino acid standard (A9906; Sigma-Aldrich, Auckland, New Zealand), two QC’s then the samples with a QC every 9^th^ slot to finish with a final amino acid. Analyses were conducted using a Thermo LC–MS system (Thermo Fisher Scientific, Waltham, MA, USA) consisting of a Dionex UltiMate 3000 UHPLC system coupled with a high-resolution Q Exactive Focus Quadrupole-Orbitrap mass spectrometer utilising heated electrospray ionisation to run in both positive and negative modes.

Thermo-derived raw files from C18 and HILIC (positive/negative) modes were converted to mzXLM (MSConvert),^36^ processed in MZmine^37^ for peak detection and alignment, and further refined in XCMS Online “xcmsonline.scripps.edu” for exploratory analysis. Mode-specific parameters were optimised for *m/z* deviation, peak width, noise, and signal-to-noise threshold to maximise feature detection. Background variability was reduced by removing chromatogram features with *p* > 0.05 or elevated in blanks (QC vs blank t-test). The resulting data matrices i.e., m/z, retention time, ion intensity were imported into MetaboAnalyst 6.0^38^ for quality control, removal of variables with high percent relative standard deviation (RSD) > 30% and auto-scaling. Gaussian distribution of variables was confirmed, ensuring comparability of feature intensities prior to statistical analysis.

A principal component analysis (PCA) was conducted to explore chemical composition and metabolite differences between two moringa provenances. Pathway enrichment analysis was conducted in MetaboAnalyst 6.0 for identified chemical classes and metabolite sets disproportionately represented among the detected allelochemicals relative to KEGG and HMDB background libraries. Overrepresentation was quantified using enrichment ratios and assessed for significance via Fisher’s exact test with false discovery rate (FDR) correction (*p* < 0.05), enabling detection of dominant biosynthetic categories with potential ecological roles in allelopathic interactions.

### 2.3. Allelopathic screening using root extracts method on steel blue germination paper

To investigate the allelopathic effects of MRE, a three-factor factorial experiment was established using a completely randomised design (CRD). The factors included (i) two moringa provenances: provenance-t and provenance-g; (ii) six extract concentrations, i.e., 0% (control, distilled water), 1%, 10%, 20%, 50%, and 100% (w/v); and (iii) five target species, including mānuka, ryegrass, white clover, fathen, and crabgrass, and five biological replications were used for each treatment. These concentrations were selected based on results from preliminary experiments carried out prior to the main study. These preliminary tests were used to identify dilution levels that generated detectable allelopathic responses without causing complete germination failure, ensuring that the final concentration range captured both sub-inhibitory and inhibitory effects.

Aqueous root extracts were prepared by homogenising fresh roots collected from 120-day-old moringa plants grown under glasshouse conditions at temperatures ranging from 24–27 °C (±3 °C), while light and relative humidity were not controlled. The roots were soaked in double distilled water for 24 hours, followed by double filtration to obtain the working concentrations (w/v). To prepare the extract dilutions, a stock solution of MREs was first produced by homogenising a known mass of fresh root tissue in a defined volume of distilled water i.e., 1:10 w/v followed by filtration to obtain a liquid extract. This stock was treated as 100% extract, representing the undiluted filtrate. All subsequent concentrations (0−100%) were prepared by diluting the stock extract with distilled water.

For each treatment, the top-of-paper method^39^ was used, where 10 mL of each extract concentration was applied uniformly to Steel Blue Germination Paper (Anchor Paper Company, St Paul, MN), which was placed in plastic containers (15 × 12 × 10 cm, PS Limited, Auckland, New Zealand). Fifty seeds of each target species were placed on germination paper in a container and sealed with a lid before being placed into a controlled growth unit (Conviron Ltd., Canada). The germination conditions were set at 20/25 °C for white clover and mānuka and 20/30 °C (day/night temperatures) for perennial ryegrass, fathen, and crabgrass. Germination was scored as radicle emergence (RE) (2 mm) every 4 h for ryegrass and white clover. The final germination count (normal seedlings) was determined seven days after sowing.^39^ For mānuka, fathen, and crabgrass, RE was observed every 24 hours, and the final count was performed 21 d after sowing. These observation periods were selected based on the results from preliminary experiments carried out prior to the main study. Germination (%) was calculated on the basis of the final count of normal seedlings (seedlings without germination defects) as a fraction of the total number of seeds planted multiplied by 100. The mean germination time (MGT) was calculated following the formula of Ellis and Roberts^40^:

$$\mathrm{MGT}=\frac{\sum (\mathrm{ni}\cdot \mathrm{ti})}{\sum \mathrm{ni}}$$

where *n*_*i*_ = the number of seeds that germinated on day *t*_*i*_, ti = the number of days from the beginning of the experiment, and ∑ni = the total number of seeds that germinated at the final count.

### 2.4. Pot based plant–plant interference assay for screening moringa root allelochemicals

Besides the use of MREs, we assessed the allelopathic potential of moringa root allelochemicals through a pot-based assay following the procedures of Wu et al.,^41^ with minor modifications. Briefly, a single moringa plant and fifty seeds of each target species were co-cultivated in 12.5 cm diameter pots with seeds sown in a circular pattern around the moringa plant. Each pot was filled with 1500 g of sterilised sand and compost mixed at a ratio of 1:2 (v/v) to serve as the growth medium. The target species were introduced approximately 120 d after moringa was established to ensure full root distribution within the pots. A bioassay ring (10 cm inner diameter, 2 cm height) was gently pressed onto the pot surface, leaving the moringa litter layer undisturbed. Within each ring, fifty surface-sown seeds of the target species (one species per pot per time) were evenly spaced, lightly covered (≈2 mm) with the same potting substrate, and sieved through a 2 mm mesh to standardise seed‒soil contact. Pots without moringa plants were included as positive control treatments.

The target species were mist irrigated daily with deionised water, and no fertiliser was applied during the experiment. The germination percentage (radicle ≥ 2 mm) was recorded 7 d after sowing (DAS) for white clover and ryegrass,^39^ 21 DAS for fathen and crabgrass, and 28 DAS for mānuka (DAS for each species were selected from preliminary experiments). At 10 DAS, five randomly selected white clover and ryegrass seedlings per ring were gently excavated, and their root and shoot lengths (cm) were measured using digital callipers; at 22 DAS, the same measurements were taken for crabgrass and fathen; and at 29 DAS for mānuka. The remaining white clover and ryegrass seedlings were harvested at 12 DAS, 22 DAS for crabgrass and fathen, and 30 DAS for mānuka, oven dried at 70 °C for 72 hours and weighed separately to determine shoot and root biomass (g).

### 2.5. Statistical analyses for response species

To determine the concentration effects of the MRE, data were subjected to general linear models (GLMs) to account for potential non-normal distributions in the response variables using R-statistical package ver. 4.4.2.^42^ Fixed effects included variety, concentration, species, and their interactions; whereas replicates were treated as random effects to account for variability across experimental runs. The significance of fixed effects was tested using Type III fixed effects, which evaluate the significance of each factor after accounting for all other fixed effects and their interactions in the model, and treatment effects were evaluated relative to the control treatment (distilled water). When the interaction was significant, the concentration effect was probed by comparing the concentration effect within each species using Tukey‒Kramer-adjusted least-square means (LSMeans) (*p* < 0.05), and estimates are presented as LSMeans ± SEs.

For the pot-based plant‒plant interference experiments, statistical analysis was performed using one-way ANOVA to test for significant differences among the treatments, and mean separation was conducted with Tukey’s HSD test (*p* < 0.05).

The allelopathic potential of the aqueous root extracts and root exudates was determined in terms of germination, root and shoot length, and root and shoot biomass. The degree of inhibition or stimulation was determined by comparing the growth responses of the seedlings of the target species to those of the control plants via the allelopathic response index (ARI) via the following equation according to Wang et al.^43^:

ARI=1−C/T(T≥C),RI=1−T/C(T<C)

where T represents the data from the treatment variables, C represents the data of the control group, ARI < 0 represents an inhibitory effect and ARI > 0 represents a stimulating effect. A heatmap was used to determine the extent of the allelopathic effect, i.e., deep red (RI) ≤ −0.50 indicates strong inhibition, whereas deep blue (ARI) ≥ 0.25 indicates a potential stimulatory effect on the target species.

## 3. Results

### 3.1. Metabolite profiling and allelochemical identification in moringa provenances

**Figure 1.**
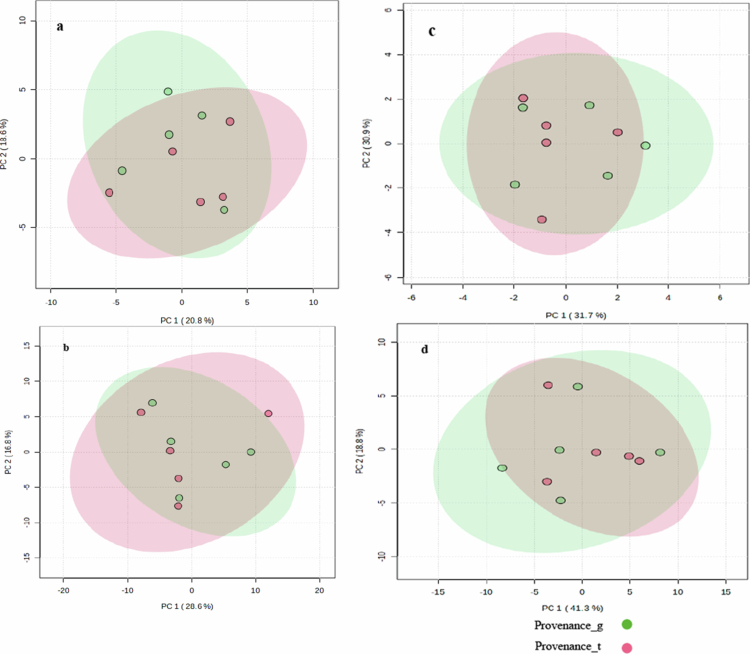
Principal component analysis (PCA) score plots comparing the metabolite profiles of two moringa provenances under different ionisation modes: a) C18 positive (F = 0.5973, *R*^*2*^ = 0.069, *p* = 0.5410), b) C18 negative (F = 0.05762, *R*^*2*^ = 0.0720, *p* = 0.9470), c) HILIC positive (F = 0.8129, *R*^*2*^ = 0.0922; *p* = 0.4570), and d) HILIC negative (F = 0.2646, R^2^ = 0.0327, *p* = 0.5745). The 95% confidence ellipses show partial overlap between the provenances in all ionisation modes, which indicates a high degree of similarity in their metabolomic profiles. The minor variation observed may reflect environmental influences from their original provenances. PERMANOVA (999 permutations) confirmed that the differences between groups were not statistically significant (*p* > 0.05).

Principal component analysis (PCA) revealed considerable overlap in the metabolite profile of the moringa provenances (g and t) without clear clustering and/or significant separation along the principal component axes (Figure 1). The 95% confidence ellipses for both provenances converged showing high similarity in metabolite composition. Consistently, permutational multivariate analysis of variance (PERMANOVA) confirmed that the differences in metabolite profiles between the two moringa provenances were not statistically significant (*p* > 0.05).

A total of 3167 molecular features (metabolites) were detected in the MREs across the four ionisation modes of C18 and HILIC (positive and negative), of which 52 were successfully annotated based on MS/MS fragmentation and spectral-library matching, while the remaining 3115 remained unidentified. Annotated metabolites were considered to exhibit allelopathic interactions when MREs showed a significant, concentration-dependent inhibitory effect on germination or early seedling growth, demonstrated consistently across both moringa provenances and relative to the control treatment (distilled water). These compounds were dominated by phenylpropanoids and benzenoids, with smaller contributions from organic acids, oxygenated compounds, and organoheterocyclics. Among the 52 metabolites, 36 were annotated with level three identification confidence (putatively characterised on the basis of spectral similarity; see supplementary Table 1), whereas 16 metabolites achieved level two confidence confirmed by spectral library matching (Table 1).

**Table 1.** Identified allelopathic metabolites from moringa root extract with level 2 confidence based on LC-MS/MS spectral library matching.

Class/sub/sup class	Compound name	KEGG	Stream	m/z	rt.	Fragments ions ()
Cinnamic acids	M-trans-cinnamic acid	–	C18 +	163.0751	8.30	132.0507, 103.0530
Hydroxycinnamic acids	Caffeic acid	C01481	C18 +	181.0492	7.79	163.0373
Flavonoids	Naringenin	C00509	C18 +	273.0752	8.89	123.0421, 119.0455
Coumarins	4-Hydroxycoumarin	C20414	C18 +	163.0387	7.45	65.0367
Flavonoids	Isoquercetin	C05623	C18 +	465.1021	9.17	303.0433, 304.0470
Flavonoids	Epicatechin	C09728	C18 +	291.0857	8.31	139.0362
Coumarins	Umbelliferon	C09315	C18 +	163.0387	7.45	65.0077
Phenylpropanoids	Coumarin	C05851	C18 +	147.0438	8.30	103.0519
Methoxyphenols	Homovanillic acid	C05582	C18 +	183.0649	11.49	153.0541, 107.0486
Flavonoids	Naringin	C09789	C18+	581.1855	9.13	563.1536
Flavonoids	(−)-Epicatechin	C09727	C18 –	289.0711	8.28	245.0879
Alpha-Amino acids	L-Phenylalanine	C00079	Hilic +	166.086	14.21	121.084
N-acyl-alpha amino acids	Indole-3-acetyl-L-tryptophan	–	C18 +	362.1496	8.25	344.1028
Alpha-Amino acids	L-Tryptophan	C00078	Hilic +	205.0968	14.65	188.0687
Dihydrochalcones	Aspalathin	–	Hilic –	451.1236	7.47	332.0806
Carboxylic acids	Glutaric acid	C00489	Hilic –	131.0339	15.36	113.0272

Values in parentheses refer to the fragment ions (m/z) used to support identification of each annotated feature that followed the level 2 criteria of the metabolomics standards initiative (MSI), m/z = mass‒charge ratio, rt = retention time, + = positive, – = negative.

### 3.2. Enrichment analysis of identified allelochemicals in moringa provenances

**Figure 2.**
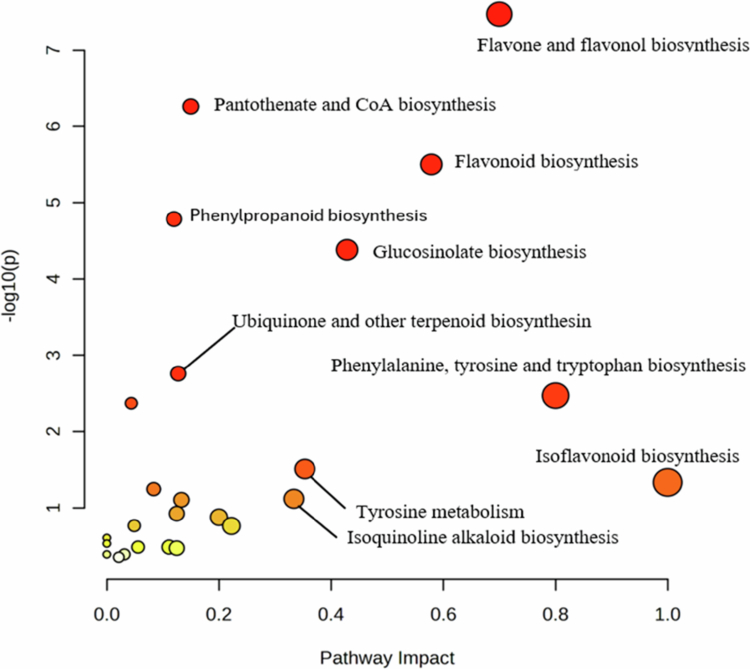
Pathway enrichment analysis of moringa root metabolites revealed significant flavonoid/isoflavonoids biosynthesis; phenylalanine, tyrosine and tryptophan biosynthesis; and flavone and flavonol biosynthesis, driven by phenolic acids, flavonoids, and coumarins.

The enrichment of bioactive chemical compounds was evaluated by quantifying feature abundance relative to the control (blanks) treatments, which served as the baseline for determining enriched metabolites. Pathway enrichment analysis of the moringa root allelochemicals revealed high isoflavonoids biosynthesis (0.97); significant enrichment for phenylalanine, tyrosine, and tryptophan biosynthesis (0.79), and a strong contribution from flavone/flavonol and broader flavonoid biosynthesis (0.69). Other significantly enriched pathways included glucosinolate, flavonoid, and tyrosine metabolism and CoA biosynthesis (Figure 2). These pathways are driven by hydroxycinnamic acids (caffeic, ferulic, and 4-methoxycinnamic acids), flavonoids and glycosides (quercetin, kaempferol, and luteolin), coumarins (scopoletin and umbelliferone), and indolic derivatives (DOPA and indole-3-carboxylic acid).

### 3.3. Effects of moringa root extracts on the germination and growth of target plant species

**Figure 3.**
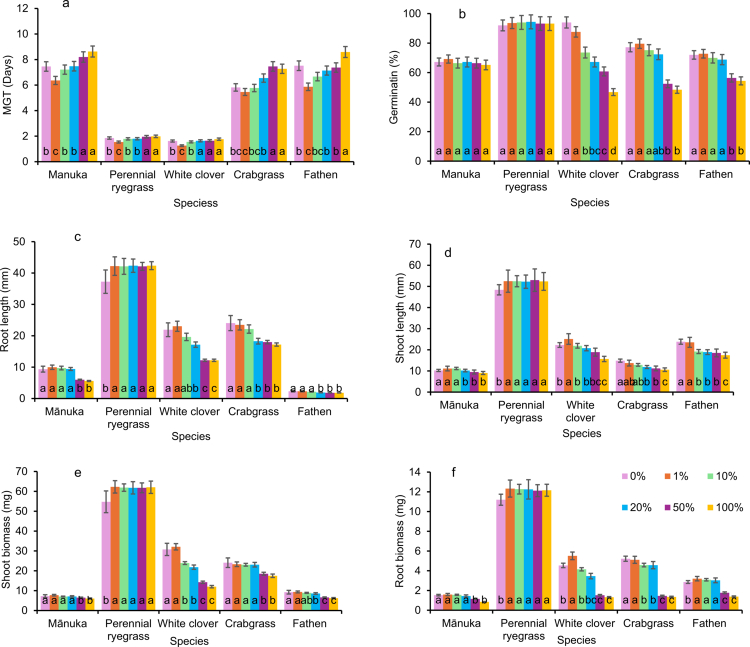
Effects of different concentrations (0, 1, 10, 20, 50, or 100%) of MRE on the germination and seedling growth traits of five target species using GLMs ‘within each species’ comparisons of a) MGT, b) germination, c) root length, d) shoot length, e) root biomass, and f) shoot biomass. Different letters inside the bars denote significant differences among concentration gradients for each species (*P* < 0.05). The values represent the LS means ± SE, with 5 replicates per treatment.

The combined concentration effect analyses indicated that germination and seedling growth traits varied significantly among the target species in response to increasing extract concentrations (Figure 3a−f). Although germination decreased with increasing extract concentration, the magnitude of inhibition was strongly species-specific and concentration dependent. Perennial ryegrass maintained high germination percentages associated with reduced MGT across all the extract concentration gradients.

In contrast, white clover, crabgrass, and fathen were sensitive to allelochemicals, with germination declining continuously from the 10% concentration, falling below 50% at the full-strength extract (100%) and subsequently increasing the MGT. Mānuka displayed an intermediate response with increased MGT at a 100% concentration. The germination and seedling growth traits at relatively low extract concentrations (1–10%) did not significantly differ from those in the control treatment for most target species. However, the inhibitory effects became increasingly visible at higher concentrations (20–100%) in more sensitive species, such as white clover.

Type III fixed effects ANOVA revealed significant interspecific variation in the responses of target species to moringa allelopathic treatments, which indicated that each species exhibited a distinctive sensitivity profile to the applied extract concentrations. The allelochemical concentration, target species, and their combination significantly influenced seed germination, mean germination time (MGT), shoot and root length, and shoot and root biomass (*p *< 0.05). However, the effects of moringa provenance alone or in combination with other factors were not significant (Table 2).

**Table 2.** Type III tests of the fixed effects of mean germination time (MGT), germination, root length, shoot length, and root and shoot dry biomass were performed, with the fixed factors of species, provenance, concentration, and their interaction.

Effect	MGT		Germination		Root length		Shoot length		Root biomass		Shoot biomass	
	F value	*p* value	F value	*p* value	F value	*p* value	F value	*p* value	F value	*p* value	F value	*p* value
**S**	**6808.03**	**<.001**	**614.67**	**<.001**	**29434.2**	**<.001**	**31276.6**	**<.001**	**6613.56**	**<.001**	**31300.7**	**<.001**
P	0.01	0.9324	0.51	0.4348	0.1421	0.9918	0.21	0.9054	0.18	0.6696	0.18	0.6722
**C**	**191.73**	**<.001**	**237.35**	**<.001**	**863.56**	**<.001**	**168.42**	**<.001**	**238.56**	**<.001**	**276.94**	**<.001**
S x P	0.21	0.9309	0.82	0.6149	0.5413	0.7041	0.25	0.9054	0.65	0.6293	0.06	0.9937
**S x C**	**23.69**	**<.001**	**47.93**	**<.001**	**117.56**	**<.001**	**68.94**	**<.001**	**46.63**	**<.001**	**163.93**	**<.001**
P x C	0.90	0.4820	0.34	0.8428	0.47	0.7962	0.14	0.9831	0.52	0.7616	0.06	0.9973
S x P x C	1.43	0.1271	1.32	0.1639	0.45	0.9801	0.15	1.0000	0.36	0.9951	0.09	1.000

S = species, P = provenances, C = concentration, S × P = species × provenance, S × C = species × concentration, P × C = provenance × concentration, and P × S × C = provenance × species × concentration interactions; Target species: perennial ryegrass, white clover, fathen, crabgrass, and mānuka; Moringa provenances: provenance_t from Tamil Nadu and provenance_g from the villages of Deoria district, Uttar Pradesh, India; and Concentration gradients: 0, 1%, 10 20, 50, and 100% (w/v). Values represent Type III ANOVA results: F = test statistic for the fixed effect; significance level was determined at *P* < 0.05.

The germination and seedling growth data were used to assess the allelopathic response index (ARI) to quantify the allelopathic activity of MRE. Crabgrass, fathen, and white clover showed progressive inhibition with increasing extract concentration, whereas perennial ryegrass exhibited a slight stimulatory effect across concentration gradients (Table 3). At low concentrations (1–10%), MRE enhanced germination and reduced MGT in most species. However, concentrations above 20% led to moderate to severe suppression of germination and a corresponding increase in MGT. Perennial ryegrass recorded positive ARI values (0.001−0.100), indicating a mild stimulatory influence on both germination and seedling growth. In contrast, concentrations between 20% and 100% induced significant inhibitory effects on all the traits except perennial ryegrass, whereas mānuka shifted toward inhibition in MGT, root biomass, and shoot length at relatively high concentrations.

**Table 3.** Allelopathic response indices (ARI) of various species in response to different MRE concentrations.

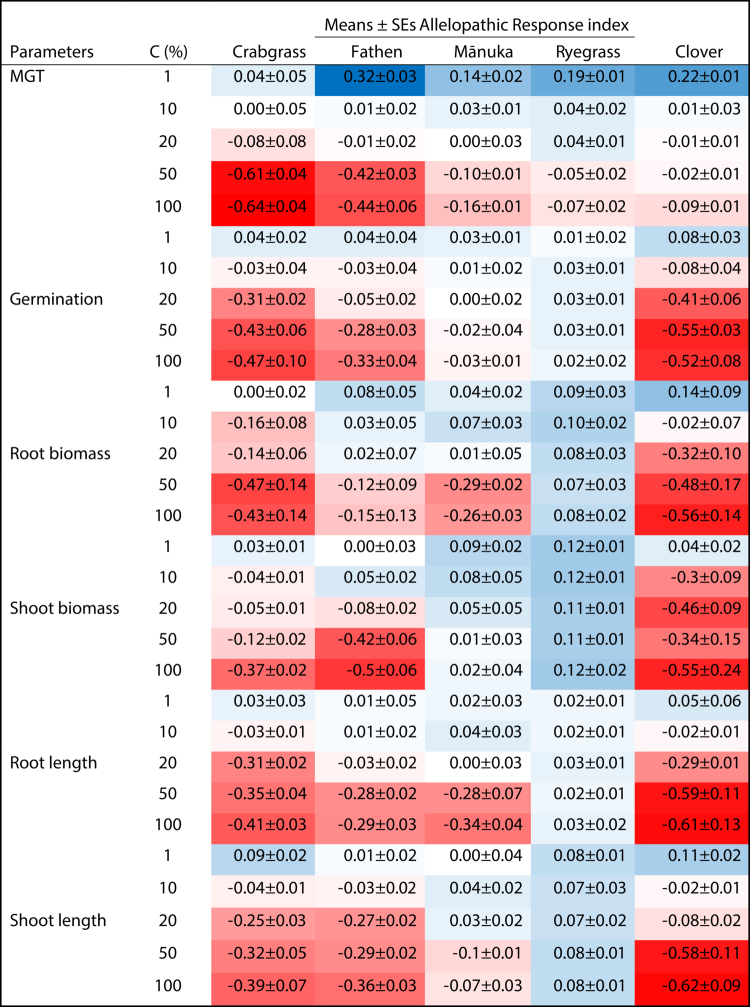

C = concentration, deep red ≤ −0.50 indicates strong inhibition, whereas deep blue ≥ 0.25 indicates a potential stimulatory effect on target species. The values represent the LS means ± SE, with 5 replicates per treatment.

### 3.4. Plant‒plant interaction effects on germination and seedling growth

Assays using target seeds planted in the same soil as potted moringa plants also revealed significant variations in germination and seedling growth among response species (see supplementary Figure 1a−e). Perennial ryegrass and mānuka seed germination was not significantly affected. However, their root and shoot lengths and root and shoot biomass were significantly enhanced compared to the control treatment. In contrast, crabgrass, fathen, and white clover showed strong allelopathic inhibitory effects, with significant reductions in germination and suppressed root and shoot development (*p* < 0.001). Both moringa provenances (t and g) followed similar response patterns without significant differences between provenances in terms of their germination and seedling growth traits.

The ARI analysis further revealed species-specific effects on the plant‒plant interactions between moringa and the target species. Crabgrass and fathen showed inhibitory responses across all the evaluated growth parameters, with the strongest suppression observed for root biomass, whereas white clover displayed significant reductions in root length and accumulation of biomass. In contrast, mānuka showed neutral to stimulatory response, as revealed by positive ARI values across all growth parameters in shoot length and root biomass, indicating a potential growth stimulation effect. However, tolerance to allelopathic effects was observed in perennial ryegrass, where responses were minimal. These allelopathic patterns indicate that MREs exert differential allelopathic pressure across species, and such species-specific inhibition against key pasture legumes and selected weed species could manifest under natural field conditions.

Table 4 present ARI values derived from the in-pot plant-plant interaction experiment, where moringa plants released allelochemicals directly into the shared substrate that allowed both chemical and rhizosphere-mediated interactions to influence target species performance. These ARIs are stronger than those observed in the extract-based assays in Table 3 which reflect the combined effects of continuous root exudation and longer exposure duration. However, both experiments showed similar species-specific trends, with strong inhibition in white clover, fathen, and crabgrass, and neutral to mildly stimulatory responses in mānuka and perennial ryegrass. Nevertheless, the magnitude of inhibition was greater in the in-pot system than in extract-based assays. While root extracts capture concentration-dependent allelopathic effects, the in-pot assay more closely reflects the cumulative allelopathic pressure likely to occur under natural filed conditions. Most importantly, despite differences in assay type, neither experiment detected significant provenance-specific differences in allelopathic potency.

**Table 4.** Allelopathic response indices (ARIs) of crabgrass, fathen, mānuka, ryegrass, and white clover to the root allelochemicals of two moringa provenances in an in-pot plant-plant interaction experiment.

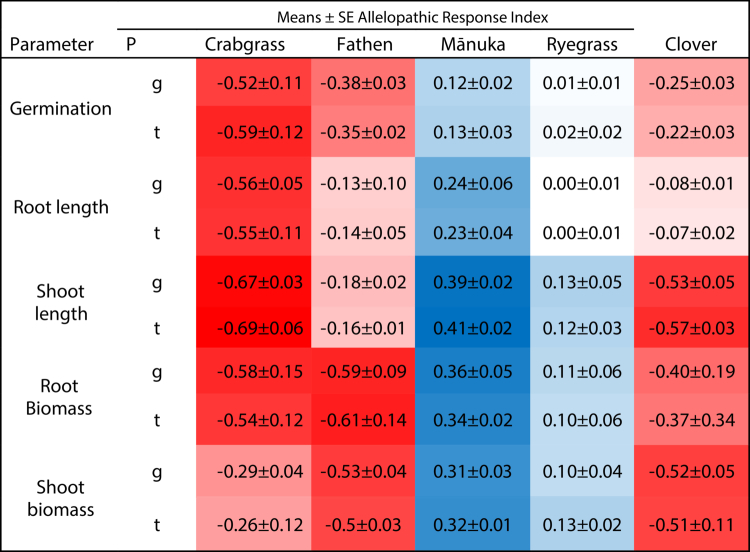

P = provenances, deep red means the response index is ≤ −0.5 (signalling strong inhibition), and deep blue means ≥ 0.3 (potential promotion/stimulation effects).

## 4. Discussion

The results of the present study revealed that moringa root allelochemicals have biological effects on other plants in a species-specific and concentration-dependent manner. The differential response to allelochemical concentrations among target species supports biochemical interactions between plants, which are mediated by the release of secondary metabolites in naturally competitive ecosystems.^12^ Germination and seedling growth suppression at relatively high concentrations (50–100%) concur with the dose-dependent principle in which the phytotoxic effect intensifies with increasing concentrations.^14^ In the context of New Zealand’s pastoral systems where perennial ryegrass and white clover form a cornerstone of productive pastures,^44^^,^^45^ the introduction of moringa could shift the competitive balance among species, potentially altering plant community composition in this agroecosystem.

The highly significant interaction effect between species and concentration confirmed that species respond differently to allelopathic stress.^12^ These interactions further indicate varying sensitivities or tolerances that may influence competitive hierarchies and species coexistence.^46^ Certain species may exhibit detoxification mechanisms or protective root exudates that may buffer the effects of allelochemical inhibition, hence conferring a competitive advantage under allelopathic stress.^47^ Among the treatments, perennial ryegrass presented the highest germination rate and the most vigorous seedling traits. This may indicate an inherent tolerance to allelochemicals through root-mediated detoxifying mechanisms or by microbiome interactions, as proposed by Scavo et al.^48^ In contrast, the growth of white clover, fathen, and mānuka were significantly suppressed at relatively high extract concentrations, indicating increased sensitivity or reduced tolerance to one or more bioactive compounds found in moringa roots.

The sensitivity of white clover in this study aligns with the vulnerability of legumes to phenolic acids and flavonoids, which disrupt root physiology and nodulation at relatively high concentrations.^49^ In contrast, perennial ryegrass exhibited resilience despite exposure, which may reflect inherent structural adaptations traits such as stronger cell wall integrity or root exudation patterns that could hypothetically buffer allelochemical stress.^50^ This selective inhibition could bias species composition towards ryegrass dominance at the expense of clover, with downstream consequences for nitrogen fixation and long-term soil fertility in pastoral systems.

The allelopathic capacity of moringa has traditionally been attributed to flavonoids and phenolic acids from leaf extracts.^51^ However, metabolomic profiling combined with pathway enrichment analysis has started to unveil how specific chemical classes of root allelochemicals may function in ecological interactions.

The absence of significant separation between provenance_g and provenance_t in the PCA as shown in Figure 1, demonstrates the high degree of similarity in their allelochemical profiles. This chemical convergence likely stems from genotypic similarities between the two provenances and emphasises the central role of intraspecific genetic relatedness in shaping the expression of allelochemicals involved in plant‒plant chemical interactions.^52^^,^^53^ Since plants were grown under the same conditions, it is not surprising that no differences were observed. However, future studies should investigate how moringa root allelochemicals respond to environmental variation.

Several allelochemicals, including phenolics (ferulic, caffeic, and chlorogenic acids), flavonoids (apigenin, naringenin, and isoquercetin), and coumarins (umbelliferone and 4-hydroxycoumarin), were annotated in moringa roots. These compounds are known to impede germination and initial seedling development by altering hormonal signalling pathways and redox homeostasis in susceptible plant species.^13^^,^^14^ In this study, the decline in germination and seedling traits across most target species (except ryegrass) highlights the inhibitory nature of allelochemicals that disrupt hormonal regulation, cell elongation, and impaired nutrient mobilisation.^51^^,^^54^ The reduction in root and shoot length could be attributed to the presence of phenylpropanoid derivatives, which are known to interfere with essential cellular processes, including reactive oxygen species (ROS), homeostasis, and auxin transport, leading to oxidative stress and reduced cell elongation.^10^^,^^55^

The enrichment of the phenylpropanoid and flavonoid pathways highlights their functional role in mediating the observed interspecific variability in growth inhibition. Hydroxycinnamic acids and flavonols derived from these pathways can disrupt amylase activity during germination, auxin transport, and cell wall expansion in young seedlings.^56^^,^^57^ These mechanisms constrain radicle emergence and seedling establishment, as observed in white clover in both laboratory and glasshouse experiments. L-Phenylalanine and caffeic acid are the central intermediaries in the phenylpropanoid biosynthesis pathway, which regulates the production of phenolics that serve as allelochemicals.^58^ These pathways may explain the strong suppression and inhibition of germination and seedling growth in white clover, fathen, and crabgrass and the lack of measurable effects on ryegrass and mānuka, further revealing species-specific allelopathic interactions. Compounds enriched in coumarin, benzoic, and indolic metabolism are known to inhibit germination and root elongation in small-seeded annuals.^59^

Pathway analysis further revealed that moringa synthesises glucosinolates widely reported in moringa tissues.^60^^,^^61^ The annotation of 1-isothiocyanato-9-(methylsulfinyl)-nonane in root extracts supports the presence of bioactive isothiocyanates generated through myrosinase-mediated hydrolysis, compounds that are known to exert phytotoxic and signalling effects in other related species such as brassicales.^62^ Beyond glucosinolates, significant enrichment of flavonoids, flavanol, isoflavonoid pathways concur with previous studies in which moringa accumulates diverse phenolic acids (e.g., caffeic, chlorogenic, p-coumaric) and flavonoid glycosides (quercetin, kaempferol), all of which are implicated in allelopathic interference and hormonal modulations.^11^^,^^63^ Additional enrichment of terpenoid, tyrosine, and isoquinoline-alkaloid pathways suggests contributions from broader secondary metabolites classes, which aligns with prior evidence of complex phytochemical defence in moringa.^13^^,^^14^ Henceforth, the glucosinolates, isothiocyanates, phenolic acids, and flavonoids identified in the present study provide a coherent biochemical basis for the inhibitory effects observed in the root-extracts and in-pot assays, which may support a multi-compound allelopathic mechanism.

In addition to direct phytotoxicity, the enrichment of these compounds may contribute to the competitive exclusion of some species through micronutrient sequestration. For example, several phenylpropanoid derivatives exhibit strong chelating affinities for essential metals such as Fe^3+^ and Zn^2+^​​​​​.^64^^,^^65^ This may limit nutrient uptake in cooccurring species such as white clover, causing a detrimental effect on seedling growth. Given the high nutrient demands of white clover (for nitrogen fixation), fathen (fast-growing annual species), and crabgrass (competitive C_4_ grass),^66-68^ micronutrient limitation could be a significant contributor to the observed growth suppression. Moringa, through allelopathic activity, could further disrupt perennial ryegrass–white clover associations that underpin pastoral productivity and alter the balance between native and introduced species.^16^ These chemically mediated interactions demonstrate that while moringa has potential as a beneficial crop, it may have negative impacts on native and pastoral species emphasizing the complex ecological effects of introducing alien species on native and agricultural ecosystems.^69-71^

## 5. Conclusion

Moringa root allelochemicals elicit concentration-dependent and species-specific effects on other native and introduced plant species. White clover, a key pasture species in New Zealand, was the most negatively affected among the tested species. Hence, the utilisation of moringa within clover pastures may not be recommended. Perennial ryegrass, on the other hand, showed no significant response to root extracts or exudates, making it more suitable for mixed cropping. Moringa was also observed to have an inhibitory effect on some common weeds, which may add value to its use in some farming systems. The enrichment analysis indicated that moringa allelochemicals are dominated by phenylpropanoids, benzenoids, and related aromatic derivatives that can influence biosynthesis pathways, transport mechanisms, and micronutrient competition. The identification of these allelopathic compounds highlights the importance of evaluating moringa not only as a crop species but also as a potential ecological driver that requires integrated management strategies to balance its agronomic value with potential risks to biodiversity and ecosystem function. Further studies should prioritise the isolation of individual compounds and further testing on pastoral, weed, and native species across different environmental contexts to disentangle their specific roles in plant growth and plant population dynamics and explore their potential uses in agriculture.

## Supplementary Material

Supplementary_information_Allelopathy-clean.docx Supplementary_information_Allelopathy-clean.docx
