# Supplementary material for: Root-derived allelochemicals from Moringa oleifera regulate germination and early seedling growth in New Zealand pasture, native, and weed species
Source: Plant Signal Behav. 2026 Mar 17;21(1):2644120. doi: 10.1080/15592324.2026.2644120 (PMC13003897; doi:10.1080/15592324.2026.2644120)
Supplement: Supplementary_information_Allelopathy-clean.docx [file KPSB_A_2644120_SM4145.docx]

**Supplementary information:**

**Supplementary Table 1.** List of 37 annotated potential allelopathic metabolites from moringa root extracts whose level 3 identification confidence was determined via LC‒MS/MS analysis.

| **No.** | **Class/Sub/super class** | **Compound Name** | **KEGG** | **Stream** | **m/z** | **rt.** |
| --- | --- | --- | --- | --- | --- | --- |
| 1 | Cinnamic | Trans-Cinnamic acid | C00423 | C18 + | 149.0595 | 2.34 |
| 2 | Cinnamic | Trans-Ferulic acid | C01494 | C18 + | 195.1126 | 7.95 |
| 3 | Cinnamic | 4-Methoxycinnamic acid | - | C18 + | 179.0701 | 8.3 |
| 4 | Phenols | Salicylic acid | C00805 | C18 + | 139.0387 | 8.85 |
| 5 | Phenol | Syringic acid | C10833 | C18 + | 199.0656 | 8.85 |
| 6 | Phenol | 4-Hydroxybenzoic acid | C00156 | C18 + | 139.0387 | 8.85 |
| 7 | 7-hydroxycoumarins | 6-Methoxy-7-hydroxycoumarin | - | C18 + | 193.0492 | 8.15 |
| 8 | Flavanol c. | Quercetin | C00389 | C18 + | 303.0493 | 9.17 |
| 9 | Flavonoid | Kaempferol-3-glucoside | C12249 | C18 + | 449.1073 | 7.36 |
| 10 | Flavonoid | Kaempferol | C05903 | C18 + | 287.0544 | 9.49 |
| 11 | Flavonoid | Luteolin | C01514 | C18 + | 287.0544 | 9.49 |
| 12 | Isoflavonoids | Genistein | C06563 | C18 + | 271.0596 | 8.44 |
| 13 | Isoflavonoids | Daidzein | C10208 | C18 + | 255.0647 | 9.2 |
| 14 | Quinolines | 4-Hydroxyquinoline | C19434 | C18 + | 146.0598 | 9.66 |
| 15 | Hydroxy fatty acids | 3-Hydroxy-3-methylglutaric acid | C03761 | C18 + | 163.0598 | 8.16 |
| 16 | Flavones | Apigenin | C01477 | C18 + | 271.0596 | 8.44 |
| 17 | Phenol lipids | (+)-Costunolide | C09382 | C18 + | 233.1532 | 8.93 |
| 18 | Indole carboxylic acids | Indole-3-carboxylic acid | C19837 | C18 + | 166.0347 | 8.05 |
| 19 | Benzenoids | Emodin | C10343 | C18 + | 271.0596 | 8.43 |
| 20 | Benzenoids | Benzoic acid | C00180 | C18 + | 123.044 | 8.30 |
| 21 | Benzoyl derivative | 2,4,5-Trimethoxybenzaldehyde | - | C18 + | 197.0806 | 7.55 |
| 22 | Glucosinolates | 1-Isothiocyanato-9-(methylsulfinyl)-nonane | - | C18 + | 248.1124 | 7.32 |
| 23 | Flavonoid | Quercetin-3-Rhamnoside | C01750 | C18- | 447.0923 | 8.77 |
| 24 | Flavonoid | Naringenin-7-O-glucoside | - | C18- | 433.1129 | 8.46 |
| 25 | Flavonoid | Quercitrin | C01750 | C18- | 447.0923 | 8.77 |
| 26 | Flavonoid | Tricin | C10193 | C18- | 329.0613 | 8.85 |
| 27 | Flavonoid | Myricitrin | C10108 | C18- | 463.0873 | 8.46 |
| 28 | Alcohols and polyols | Pantothenic acid | C00864 | C18- | 218.1024 | 2.12 |
| 29 | Benzoic acids | 4-Aminobenzoic acid | C00568 | C18- | 136.0391 | 5.59 |
| 30 | Phenols | 2-Acetamidophenol | - | C18- | 150.0548 | 6.99 |
| 31 | Alpha-Amino acid | DOPA (3,4-Dihydroxy-L-phenylalanine | C00355 | Hilic POS | 198.076 | 2.47 |
| 32 | Phenylpropanoids | Chlorogenic acid | C00852 | C18- | 353.0869 | 6.98 |
| 33 | Alpha-Amino acid | L-Tyrosine | C00082 | Hilic POS | 182.081 | 15.05 |
| 34 | Cinnamic acids | 4-Coumaric acid | C00811 | Hilic NEG | 163.0391 | 1.96 |
| 35 | Benzenediol | Pyrocatechol | C15571 | Hilic NEG | 109.0284 | 2.17 |
| 36 | Alpha-Amino acid | L-Valine | C00183 | Hilic NEG | 116.0706 | 14.73 |

The compound annotations included the observed m/z, retention time (rt), ionisation mode, and tentative chemical classification. Identification is based on spectral similarity and accurate mass matching in accordance with the MSI Level 3 criteria.


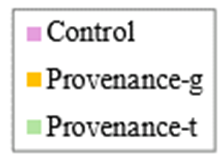


**Supplementary Fig. 1.** Effects of moringa root exudates on (a) germination, (b) root length, (c) shoot length, (d) root biomass, and (e) shoot biomass of response species in a plant‒plant pot interaction. The values represent the LS means ± SE, with 5 replicates per treatment.
